# ANNIE CAREGIVER TEXT PROGRAM: A LIFELINE IN MANAGING DEMENTIA BEHAVIORS AND STRESS

**DOI:** 10.1093/geroni/igae098.1777

**Published:** 2024-12-31

**Authors:** Jennifer Martindale-Adams, Carolyn Clark, Charles Henderson, Jessica Martin, Jennifer Roth, Linda Nichols

**Affiliations:** University of Tennessee Health Science Center, Memphis, Tennessee, United States; Lt. Col. Luke Weathers, Jr. VA Medical Center, Memphis, Tennessee, United States; Office of Connected Care, Veterans Health Administration, Washington, District of Columbia, United States; Lt. Col. Luke Weathers, Jr. VA Medical Center, Memphis, Tennessee, United States; Saint Cloud VA Healthcare System, Saint Cloud, Minnesota, United States; Lt. Col. Luke Weathers, Jr. VA Medical Center, Memphis, Tennessee, United States

## Abstract

Caring for a loved one can negatively impact well-being for caregivers and persons with dementia. The Department of Veterans Affairs’ Office of Connected Care and Caregiver Support Program expanded Annie Text Care messages to caregivers of Veterans in 2019 beginning with caregiver stress management. As of 2024, over 25,000 caregivers have enrolled in nine different caregiver subscriptions. Dementia Behaviors and Stress Management has 2,880 caregivers enrolled. For a year, caregivers receive 7 messages per week with 4 focusing on dementia behaviors and 3 focusing on stress management. Messages are educational, motivational, and activities or strategies to help based on the Resources for Enhancing All Caregivers Health (REACH) VA program. Caregivers can complete a short, Survey Monkey link, sent every 6 months. Questions are on a 5-point Likert scale ranging from completely disagree to completely agree plus open ended questions. Caregivers reported that the texts helped them manage dementia behaviors (mean score of 4.37/5), manage stress (mean score of 4.4/5), take care of themselves (mean score of 4.3/5) and their loved one (mean score of 4.5/5), and made them feel like someone cared about them (mean score of 4.6/5). Overwhelmingly, caregivers would recommend this service to other caregivers (mean score of 4.7/5). As technology moves toward increasing sophistication with artificial intelligence, this simple one-way text messaging system resonates with caregivers. In qualitative analysis themes, caregivers report that “Annie makes me feel less alone” and that messages come at the right time – “just when I needed to hear it.”

